# Online-synchronized clinical simulation: an efficient teaching-learning option for the COVID-19 pandemic time and: beyond

**DOI:** 10.1186/s41077-021-00183-z

**Published:** 2021-09-06

**Authors:** Diego Andrés Díaz-Guio, Elena Ríos-Barrientos, Pablo Andrés Santillán-Roldan, Santiago Mora-Martinez, Ana Sofía Díaz-Gómez, Joel Alejandro Martínez-Elizondo, Adrián Barrientos-Aguiñaga, Maria Nathalie Arroyo-Romero, Alejandra Ricardo-Zapata, Alfonso J. Rodríguez-Morales

**Affiliations:** 1 Education and Clinical Simulation Research Group, VitalCare Centro de Simulación Clínica, Armenia, Colombia; 2 Doctoral Program in Education, Universidad de Caldas, Manizales, Colombia; 3 Faculty of Health Sciences, Universidad Alexander von Humboldt, Armenia, Colombia; 4 Centro de Simulación Clínica - Tecnológico de Monterrey- Escuela de Medicina y Ciencias de la Salud, Monterrey, México; 5 Anesthesiology Department, Faculty of Health Sciences, Universidad Católica del Ecuador, Quito, Ecuador; 6 Grupo de Investigación Biomedicina, Fundación Universitaria Autónoma de las Americas, Sede Pereira, Pereira, Risaralda, Colombia

**Keywords:** COVID-19, SARS-CoV-2, Learning, Clinical simulation, Telesimulation, Teledebriefing, Human factors, Latin America

## Abstract

Face-to-face clinical simulation has been a powerful methodology for teaching, learning, and research, and has positioned itself in health science education. However, due to the COVID-19 pandemic, social distancing has forced universities to abandon simulation centers and make use of alternatives that allow the continuation of educational programs safely for students and teachers through virtual environments such as distance simulation. In Latin America, before the pandemic, the use of non-presential simulation was very limited and anecdotal. This article has three main objectives: to establish the efficacy of online-synchronized clinical simulation in the learning and performance of medical students on the management of patients with COVID-19 in simulation centers of three Latin American countries, to determine the quality of the online debriefing from the students’ perspective, and to deepen the understanding of how learning is generated with this methodology.

## Introduction

Clinical simulation is a teaching, learning, evaluation, and research strategy that has achieved an important place in health science education [1, 2]. This educational methodology attempts to represent reality without putting patients at risk. It is constantly developed by working with learning theories, didactics, cognitive psychology, industrial engineering, technology, and human resources [3, 4].

In these times of pandemic by the coronavirus disease 2019 (COVID-19) [5], social distancing forced universities and training centers to close their classrooms and migrate to virtual environments [6]. An estimated 1.3 billion students have withdrawn from their daily academic routines in 186 countries, including all of Latin America [7]. According to UNESCO, this represents 70% of the worldwide student population [8]. However, the pandemic has accelerated the digital transformation of medical education, allowing students to review concepts and build knowledge through webinars and other virtual strategies without having to suspend classes or expose themselves to the risk of contagion. Still, it has limitations that are perceived by students, mainly those who should already be in clinical practice, possibly affecting their motivation to learn [9].

Non-presential simulation has been developed in the last decade with terms such as remote simulation [10, 11], online simulation, which can be synchronous or asynchronous [12], telesimulation [13, 14], among others. These have shown promising results in student satisfaction, concept learning, and psychomotor skill development when there is task trainer availability; nonetheless, there are still doubts regarding the technical feasibility, the logistical aspects and the way in which learning is generated with this methodology [15].

During the COVID-19 pandemic, non-presential simulation began to be used more frequently in Latin America in order to maintain the teaching-learning processes in medical schools; nevertheless, the limitations of virtual environments can have a negative impact on the learning of medical students in low-and middle-income countries, where the technological and connectivity resources available are possibly fewer due to the existing inequality [16].

In this article, we describe the way in which we transformed the activities that we used to do in the face-to-face simulation in three Latin American simulation centers (Briefing, simulated cases and debriefing) to a synchronized online environment, and the way in which we studied it through educational research.

The main objectives of the study were to evaluate the learning and performance of the participants in the diagnosis, treatment, and non-technical skills for the case management of patients with COVID-19 during online simulation in real time. The secondary objectives were to know the satisfaction level that medical students and residents had regarding the webinar-based education they received during the pandemic and the perception of learning with an online-synchronized simulation strategy. In addition to this, we evaluate the quality of the structured debriefing from the student's perspective, as well as the simulation and debriefing times, the relationship between variables, and the comparison of the results between the three participating countries.

## Methods

### Study design

We conducted a comparative before-and-after study with mixed design, between 13 to 25 May 2020, in three Latin American clinical simulation centers (Colombia, Ecuador, and Mexico). A simulation-based educational intervention with cases related to COVID-19 was proposed in both the emergency room (ER) and the operations room (OR).

### Sample and ethics

The sample consisted of 4th, 5th, and 6th year medical students as well as anesthesiology residents who had practiced at the participating simulation centers before the COVID-19 pandemic. The students who participated in this study came from the medical school of Tecnologico de Monterrey (Mexico), Alexander Von Humboldt University (Colombia), and Pontificia Universidad Catolica del Ecuador (Ecuador). From Colombia 55 students were invited and 49 attended (89%), from Mexico 33 students were invited and 33 attended (100%), and from Ecuador 38 were invited and 24 students attended (63%).

This research was approved by the research ethics committee of the VitalCare Clinical Simulation Center with registration # CEIC-005-05-2020.

### Settings

The study was carried out in three Latin American simulation centers; in Mexico the Tecnologico de Monterrey’s center, in Ecuador the center of the Pontificia Universidad Catolica del Ecuador, and in Colombia the VitalCare Simulation Center.

### Interventions

We summarized the simulated clinical cases and the structure of the activity in Table 1.

**Table 1:** Key elements of simulation-based research [17]

Element	Descriptor
Participant orientation	Participants were guided by the course director at each center. A constructive conversation was encouraged in which the team that would be in charge of the simulation and the participants were introduced. The methodology of the course, the tools that would be used for the simulation, the learning objectives, and the investigative intention of the activity were explained. The scheduled time for this activity was 30 min.
Simulator type	We used and transmitted the data (vital signs) generated by the Laerdal® ALS® and SimMom® patient monitor.
Simulation environment	The simulations were all in online-synchronized format. The participants and the simulation staff of each center were in their homes, connected through the meeting platform on their personal computers and mobile devices.
Simulation scenario	*Learning objectives* The learning objectives were the initial diagnosis and management of the patient with COVID-19, the safe use of personal protective equipment (PPE), and the mastery of two non-technical skills (situational awareness and communication). *Team practice* The participants (students) formed teams and held positions in the care of the simulated case, these were leader, airway management, monitoring, medications, and information management. *Facilitators* All the teachers were medical specialists (internist, anesthesiologist, and intensivist) and had training in education and simulation with more than 10 years of experience. The leaders of each simulation team were the directors of the simulation centers, who were medical specialists and had training in education and simulation (ER-B and PAS-R: international courses, DAD-G: Fellowship and Doctorate) *Simulation staff* For each center an engineer was in charge of the operations area, a confederate played the role of nurse, and an actor the role of the patient participating; all of them had training in simulation (courses) and experience in the field of more than 3 years.
Instructional design or exposure	For this study, we used the sequence that we traditionally used in face-to-face simulation: Briefing, simulated case in teams, and debriefing. *Duration* We planned that the time for each simulation was not arbitrary, but should not exceed 30 min. The same applies for the time of the debriefing, the duration should be necessary to achieve a learning conversation, nonetheless, not exceeding one hour. *Assessment* The work with each team of students consisted of two clinical cases: in the first case, the students’ performance (T1) was evaluated before carrying out the first structured debriefing, where knowledge and performance gaps were closed. In the second case (T2), performance was evaluated, followed by a second structured debriefing session.
Debriefing	We used structured debriefing (with good judgment) [18], with both a debriefer and a co-debriefer. The learning conversation was conducted by the director of the simulation center in each country.

### Cases

Six simulation cases related to COVID-19 were designed, two in each country:

#### Colombia

Case 1 (T1)*:* Young woman with upper gastrointestinal bleeding due to NSAIDs. Background of mild cough, headache, unquantified fever, and contact with a patient with severe respiratory symptoms*.* Case 2 (T2)*:* Elderly male patient, with respiratory distress, cough, fever, and anosmia. Admitted to ER in shock and acute respiratory failure.

#### Mexico

Case 1 (T1): 76-year-old man with heart disease, has had a hip fracture for two weeks on treatment with Ketorolac. He was admitted to the emergency room for abdominal pain, upper gastric bleeding, and unquantified fever. Case 2 (T2): 68-year-old man, diabetic, smoker, and multiple allergies. He was admitted to the ER in acute respiratory failure and high fever.

#### Ecuador

Case 1 (T1): 32-year-old woman, 40 weeks pregnant, admitted to the operating room for respiratory failure and loss of fetal well-being. Case 2 (T2): A 39-year-old woman, 36 weeks pregnant, admitted to the operating room with placental abruption and respiratory failure.

**Fig. 1:**
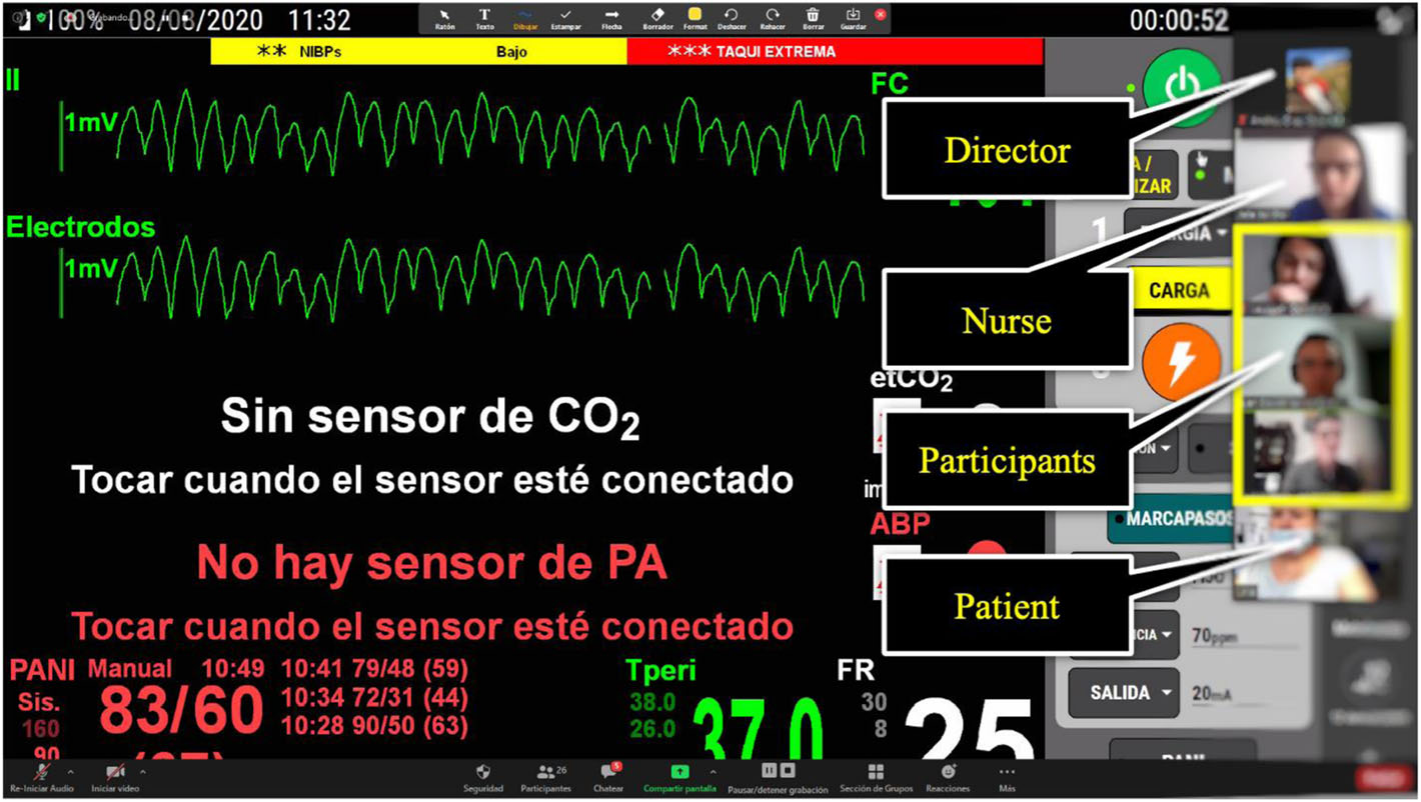
Example of elements of online-synchronized simulation

The connection was made through the Zoom® video-meeting platform (Zoom Video Communications, Inc., USA). Engineers were in charge of operating the Laerdal’s ALS® and SimMom® simulator monitor and transmitting vital signs and images. The teachers were in charge of doing the briefing, conducting the debriefing and evaluating the students. The Confederates took it upon themselves to stay in contact with the students during the simulations. Each case had a stage director who communicated with the patient and the nurse through the private zoom chat (Fig. 1). The simulated patient monitors were used, which were already present in our simulation centers prior to the pandemic.

In order to avoid the system crashing in the event of an internet connection fluctuation, several co-hosts were assigned, so that if the host left the meeting, one of the participants could remain as the new meeting’s administrator.

### Quantitative measures

#### Performance and learning behaviors

For the students’ performance evaluation during the simulated cases, we use a performance scale of 9 points (1 to 9) where a minimum rating is 1, which means that the student shows a very, very poor performance. The maximum rating is 9, which means that the student shows a very, very good performance. This scale was designed by our group, it was validated by experts to evaluate the performance of clinical teams in a previous research [19].

For the evaluation of cognitive engagement, we used the Interactive, Constructive, Active, and Passive framework (ICAP), which is based on the behaviors of individuals towards the learning activity. The participant with passive behavior receives the information without managing it, the participant with active behavior asks for information, shows interest in the task. The constructive participant reflects on the situation, contrasts the information, and the interactive participant shows high interest in the task, reflects and proposes solutions, interacts with their peers, explains the situation [20].

As previously stated, the teachers were in charge of evaluating the performance and behavior of the students, all of them were informed of the nature and objectives of the study, as well as the tools that were used to evaluate. They held synchronous meetings through the Zoom® video conferencing platform to train in the use of performance and learning behavior assessment tools.

#### Participants’ satisfaction with the online simulation

This instrument consisted of two five-point satisfaction scales for participants to rate both the online activities based on conferences (webinars) and the online-synchronized simulation received.

#### Perception of learning

We developed a Likert-type survey of 20 statements and five options (1: Totally disagree; 5: Totally agree). The items were constructed from the learning objectives of the course and the expected behaviors for the activity. This instrument included the perception of learning, teamwork, communication, and realism. We conducted a pilot of the cases and the scale, the latter was consistent, obtaining a Cronbach’s alpha of 0.73.

#### Debriefing Assessment for Simulation in Healthcare (DASH)

The Debriefing Assessment for Simulation in Healthcare (DASH)® Student Short-form scale was used to assess the quality of debriefing. This scale contains six elements that encompass the instructor’s behaviors: Introduction to the simulation environment, the engaging context for learning, organized debriefing structure, provoked reflection of performance, and identification of what was done well and poorly, which helped determine how to improve or sustain good performance. Element ratings are based on a 7-point effectiveness scale (1: Extremely Ineffective/Detrimental; 7: Extremely Effective/Outstanding) [21]. The students were instructed in its use.

### Qualitative measures

Two open-ended questions were asked for participants to express their views on the strengths (question A) and the weaknesses (question B) of the online-synchronized simulation.

#### Data collection

Performance evaluation and assessment of learning behaviors were collected in a google sheet®. The instruments of satisfaction, learning perception, quality of debriefing, and strengths and weaknesses of the synchronous online simulation were sent to the participants via Google Forms® (Google LLC, USA). The information was collected between May 13 and 25, 2020.

#### Statistics

Statistical analysis was performed in SPSS 26® (IBM, USA). The normality of the distribution of the data was evaluated with the Kolmogorov-Smirnov test, the qualitative variables were summarized with proportions, and the quantitative variables with measures of central tendency and dispersion. The comparison of qualitative variables was performed with the chi-square test. Pretest and posttest scores were compared with the Wilcoxon Test. Relationships between variables were calculated using the Spearman’s Rho correlation coefficient. Statistical significance was expressed as a function of *p* < 0.05.

#### Qualitative analysis

Atlas.Ti V8.1 (Scientific Software Development GmbH, Germany) software was used for the qualitative analysis of two open-ended questions related to the online-synchronized simulation strengths and weaknesses. We carried out a Thematic Analysis [22]. For this, we initially read (DAD-G, AR-Z) the text several times, then we made a general coding of possible themes, having the initial themes labeled with colors that corresponded to known categories, for example, teamwork, communication, learning, and realism. On the final phase, we reviewed the codes and reorganized themes: in this step, we disregarded some of the codes and regrouped the themes until the final ones were picked, which were then exported to a spreadsheet in order to be summarized as proportions.

## Results

### Sample

One hundred and six medical students, 49 from Colombia (46.2%), 33 from Mexico (31.1%), and the remaining (22.6%) from Ecuador participated in the study. Mean age was 23 years (IQR: 22–26), and (51.9%) were men. Regarding the academic level, (34.9%) were fourth year students of medicine, (38%) of fifth year and (4.7%) of 6-year (21.7%) were anesthesia residents.

### Times

Fourteen online-synchronized simulation (OSSim) sessions were performed with a total duration of 25.1 h with a mean of 102.7 min. In each session, two clinical cases were executed with their respective structured briefing and debriefing. The relationship of debriefing time with simulation time (D/S index) was 1.33. In Table 2, we presented the educational activities’ times.

**Table 2:** Educational activities time (minutes)

	Briefing 1	Case 1	Debriefing 1	Briefing 2	Case 2	Debriefing 2
**Colombia**	4.08	21.24	24.16	2.09	22.53	25.90
**México**	5.63	20.38	32.15	2.87	20.51	34.40
**Ecuador**	2.83	18.00	25.00	1.07	21.00	23.00
$\overline{\mathrm{x}}$	**4.18**	**19.87**	**27.10**	**2.01**	**21.35**	**27.77**

### Participants’ satisfaction with the online simulation

The satisfaction score for online education (webinars) during the COVID-19 pandemic was lower than that of online-synchronized simulation: 3 (IQR: 3–4) vs 5 (IQR: 4–5). A difference by country was found, being lower in Colombia for online education (*p <* 0.001), and the level of satisfaction for the online simulation was lower in Mexico (*p =* 0.021).

### Performance and learning behaviors

No difference in performance was found by sex, however, a statistically significant difference was found by educational level, being greater before and after the intervention in the anesthesia residents (*p* < 0.05). The comparison of the before and after performance is summarized in Table 3. The cognitive engagement was passive (10.4%), active (11.3%), constructive (34.9%), and interactive (43.4%).

**Table 3:** Performance in COVID-19 simulated cases 1 and 2 (*N*: 106)

	Case 1		Case 2		
Performance	Median	IQR	Median	IQR	***P*** value
Diagnosis	4	3–6	8	7–8	< 0.001
Treatment	4	3–7	8	7–9	< 0.001
Donning^a^	3	1–5	8	7–8	< 0.001
Doffing^a^	3	1–5	8	6–9	< 0.001
Awareness	4	3–6	8	7–8	< 0.001
Communication	4	4–5	7	6–8	< 0.001

Wilcoxon test

Note: ^a^Performance in donning and doffing was evaluated from the declarative aspects of knowledge

### Perception of learning

Out of the 106 participants, 100 answered the survey (94.3%). A high agreement level was found with the OSSim inventory in all of its items (Table 4). This instrument showed a good internal consistency with Cronbach’s alpha of 0.87.

**Table 4:** Agreement proportion to online-synchronized simulation (*N*: 100)

Item	Totally disagree	Disagree	Neutral	Agree	Totally agree
**1**	1.0%	4.0%	12.0%	36.0%	47.0%
**2**	0.0%	0.0%	7.0%	37.0%	56.0%
**3**	1.0%	9.0%	19.0%	28.0%	43.0%
**4**	5.0%	7.0%	4.0%	44.0%	40.0%
**5**	0.0%	0.0%	10.0%	26.0%	64.0%
**6**	0.0%	0.0%	4.0%	21.0%	75.0%
**7**	1.0%	9.0%	22.0%	30.0%	38.0%
**8**	0.0%	1.0%	4.0%	26.0%	69.0%
**9**	1.0%	3.0%	9.0%	36.0%	51.0%
**10**	1.0%	1.0%	8.0%	36.0%	54.0%
**11**	4.0%	13.0%	13.0%	31.0%	39.0%
**12**	0.0%	8.0%	4.0%	27.0%	61.0%
**13**	5.0%	6.0%	16.0%	34.0%	39.0%
**14**	0.0%	2.0%	6.0%	14.0%	78.0%
**15**	0.0%	1.0%	17.0%	35.0%	47.0%
**16**	2.0%	8.0%	11.0%	23.0%	56.0%
**17**	0.0%	1.0%	5.0%	21.0%	73.0%
**18**	4.0%	4.0%	12.0%	36.0%	44.0%
**19**	3.0%	3.0%	7.0%	36.0%	51.0%
**20**	0.0%	0.0%	2.0%	16.0%	82.0%

The questions were grouped into four categories: Realism, Learning, Non-technical Skills Training (NTS), and Active Learning Strategy (ALS). The answers were grouped into three agreement levels: low, middle, and high. The level of agreement was mainly high: Realism (88%), Learning (89%), NTS training (94%), ALS (95%).

No statistically significant difference was found for age, sex, or educational level. Difference was found by country in the perception of realism (*p=*0.030) and learning obtained (*p=*0.037), being lower in Mexico. But not at the perception of non-technical skills training (*p=*0.12) or active learning (*p=*0.8).

### Debriefing assessment

The evaluation of the debriefing’s quality was high (Table 5). No significant differences by sex or age were found. Fourth-year students and resident physicians rated element 1 higher than fifth- and sixth-year students (*p* = 0.023). In the analysis by country, the scores in Colombia were higher for element 1 (*p* < 0.001), element 2 (*p* = 0.04) and element 3 (*p* = 0.033). No statistically significant difference was found for the other elements.

**Table 5:** Debriefing Assessment for Simulation in Healthcare (DASH) (*N*: 100)

		Rating scale						
Element	Descriptor	1	2	3	4	5	6	7
**1**	The instructor set the stage for an engaging learning experience.	0.0%	0.0%	0.0%	0.0%	0.0%	22.0%	78.0%
**2**	The instructor maintained an engaging context for learning.	0.0%	0.0%	0.0%	0.0%	5.0%	13.0%	82.0%
**3**	The instructor structured the debriefing in an organized way.	0.0%	0.0%	0.0%	1.0%	2.0%	13.0%	84.0%
**4**	The instructor provoked in-depth discussions that led me to reflect on my performance.	0.0%	0.0%	0.0%	1.0%	0.0%	23.0%	76.0%
**5**	The instructor identified what I did well or poorly—and why.	0.0%	0.0%	0.0%	3.0%	4.0%	21.0%	72.0%
**6**	The instructor helped me see how to improve or how to sustain good performance.	0.0%	0.0%	0.0%	0.0%	2.0%	15.0%	83.0%

### Bivariate analysis

In the bivariate analysis, a strong positive correlation was found between cognitive engagement and the categories related to simulation-based learning, being stronger with realism (*p* < 0.001). Another correlation was found between cognitive engagement and performance, being stronger with communication (*p* < 0.001), and an intermediate positive correlation between cognitive engagement and improvement in situational awareness and treatment. In Table 6, we summarized the correlations with Spearman’s Rho.

**Table 6:** Bivariate Analysis (*N*: 100)

	Rho	***p***
**Learning**		
Learning - Cognitive Engagement	0.30	**0.007**
Learning - Realism	**0.60**	**< 0.001**
Learning - NTS Training	0.30	**0.002**
Learning - Active Learning Strategy	0.28	**0.003**
**Cognitive Engagement - Performance**		
Cognitive Engagement - Diagnosis	0.28	**0.004**
Cognitive Engagement - Treatment	0.45	**< 0.001**
Cognitive Engagement - Donning	0.30	**0.002**
Cognitive Engagement - Doffing	0.30	**0.002**
Cognitive Engagement - Awareness	0.45	**< 0.001**
Cognitive Engagement - Communication	**0.71**	**< 0.001**

Spearman’s Rho

Note: In bold, the high correlation and statistically significative differences

### Qualitative analysis

Open and selective coding of the texts written by participants (*N*: 100) was carried out. Regarding the strengths, 12 codes were found that represented the students' thinking, with 256 citations. 41% of the students highlight the realism, and 36% the social interaction. The most common code concurrences were found between perception of realism and real-time interaction, realism and theory-practice integration, and realism with the opportunity to carry out social practice (Table 7).

**Table 7:** Proportions of online-synchronized clinical simulation strengths (*N*: 100)

Realism	41.0%
Real-time interaction	36.0%
Social practice	30.0%
Theory-practice integration	20.0%
Safe learning environment	18.0%
Fixing errors	18.0%
Learning trough debriefing	18.0%
Awareness development	16.0%
Knowledge gaps diagnosis	16.0%
Know and learn to use personal protective equipment (PPE)	13.0%
Communication practice	10.0%
Performance gap diagnosis	8.0%

Regarding the weaknesses of online-synchronized clinical simulation, (36.4%) of students recognized the intermittency of communication due to the saturation of the platform when they spoke at the same time, (35%) described their dependence on internet speed, and (32.3%) considered the lack of practice of motor skills such as orotracheal intubation, donning and doffing of personal protective equipment, among others, as a limitation (Table 8). No strong concurrences were found.

**Table 8:** Proportions of online-synchronized clinical simulation weaknesses (*N*: 100)

Intermittent communication	36.4%
Internet velocity dependence	35.0%
Motor skills training absence	32.3%
Is a new experience	21.0%
Having no contact with the manikin	12.0%
Student number (high)	5.4%
Software and APP	4.0%
Nothing	4.0%

## Discussion

In the current study, the results demonstrated a low level of satisfaction from medical students with the methodology of education in virtual settings based exclusively on webinars through conference platforms. This was the dominant strategy at the beginning of the COVID-19 pandemic to maintain the processes of teaching-learning and constructing knowledge despite social distancing [8, 23]. In contrast, a high level of satisfaction with learning was found with the online-synchronized clinical simulation. The latter can be explained from the qualitative analysis of the students’ discourse, since this strategy allowed them interaction, social practice, possibility of making decisions, and integrating theory with practice. The online-synchronized simulation has characteristics that make it a social practice [24].

An interesting finding with this interactive methodology was the time needed to achieve the learning objectives. Online-synchronized simulation requires more time than we used in face-to-face simulation for the case development. The debriefing time was similar to the one we used in the simulation center. However, the debriefing and simulation relationship was lower than that found in other studies [25]. This could be due to the participants describing what they did during the online simulation, and the turns taken to speak, which lengthened the time of the simulated cases.

It is possible that clinical simulation is superior to traditional passive educational practices for developing skills and integrating learning [26–28], as it has had an essential technological advance to emulate clinical environments [29]. Nonetheless, the evidence is not conclusive that with more fidelity of the simulators, more learning is achieved [30]. Similar results were found in other studies with online simulation [12, 13].

In our study, the perception of realism was high; we think that this was due to the great social interaction in real-time with peers, standardized patients, staff, the immediate feedback shown on the hemodynamic monitoring, and the complementary diagnostic aids, which were favored by the briefing and structured debriefing.

The posttest learning levels were high, which corresponded to the perception of learning, this is perhaps more related to constructive and interactive cognitive engagement, social interaction, and the environment created by the instructors during the briefing and debriefing [31]. In a similar manner, we think that despite the fact that the second case was different from the first, maintaining the same theme and structure of the simulation (briefing ➔ simulated case ➔ debriefing) allowed greater comfort for students, which might be involved in a better performance.

Patel et al. conducted a similar study with 53 anesthesiology residents. Knowledge was evaluated with pre- and post-tests, and satisfaction with the activity through a survey. They found improvements in learning and satisfaction with simulated online activities, with the biggest downside to telesimulation was the audio quality. All of the above is consistent with our findings [32].

During this pandemic, human factors have shown to be related to risking or protecting health care workers [33–35]. The mastery of non-technical skills such as communication, awareness, leadership, and teamwork, as well as the management of the cognitive load, are both determining factors for success [19]. The level of performance in the initial evaluation (T1) was low, which is largely explained by the novelty of the disease, by the lack of knowledge of the safe technique of personal protective equipment (PPE) usage, and by the scarce formal curricular insertion of non-technical skills during the undergraduate level.

The declarative components of knowledge regarding safe airway management and correct usage of PPE, along with the mastery of communication strategies and situational awareness improved significantly on the second simulated case. This we attribute to the changes done in the conceptual model of the participant's biosafety, using checklists for donning and doffing, distributing the attention, using the strategy of “pause and think,” calling to the sterile cockpit, and improving the closed communication loop.

The online debriefing (teledebriefing) in this work obtained a very good rating from the students. We believe that this result is mainly due to two situations, the first is the experience conducting debriefing with the leaders of each simulation center, and the second is the structure carried out during the activity, since we decided to use the same dynamics that already existed and had succeeded in the face-to-face simulation prior to the COVID-19 pandemic. We managed to build a safe learning environment for the participants, and kept their interest during the course. We think that it is possible that the participants’ perception of their learning and performance was also influenced when rating the debriefing. This result is consistent with the study by Ahmed et al .[36], who carried out teledebriefing, which was evaluated using the DASH scale in the student version with satisfactory results.

### Limitations and strengths

This study has some limitations, in the design, the lack of a control group and randomization may decrease the internal validity. From a technical aspect, the dependence on the quality of the internet could be involved in the low cognitive engagement of some participants, however, the evaluation of the activity was high. A fundamental limitation was that only the declarative aspect of the procedures could be worked on. Regarding strengths, this was a multicenter and multinational study, its sample was larger than that of similar studies, and the internal consistency of the instruments used to collect the information was high.

The limitations of this work can be addressed in future studies with a multidisciplinary sample, with more countries participating, and with a performance evaluation after the online simulation is done in the simulation centers.

## Conclusion

Although the COVID-19 pandemic has promoted social distancing and online conference-based education, the level of students’ satisfaction tends to decrease. Online-synchronized simulation is an active and social learning activity that enables the training and developing of non-technical skills, as well as improving the declarative knowledge of medical students without having to increase costs or sacrificing the perception of realism by the learners, and an efficient alternative for teaching and learning in health sciences in the new normalcy. For this, it is essential to perform an adequate briefing, allocate more time for cases, and carry out structured debriefing. Having said that, it is recommendable that in a face-to-face modality the procedural aspects be complemented in the simulation centers with the appropriate biosafety protocols.

## Data Availability

The anonymized data used for the analysis of the present study are available from the corresponding author on reasonable request.

## Abbreviations

ALS: Active learning strategy
COVID-19: Coronavirus disease 2019
DASH: Debriefing assessment for simulation in healthcare
ER: Emergency room
ICAP: Interactive, constructive, active, passive framework
NTS: Non-technical skills
OR: Operations room
OSSim: Online-synchronized simulation
PPE: Personal protective equipment
